# Evaluation of the Effects of *Pinus koraiensis* Needle Extracts on Serum Lipid and Oxidative Stress in Adults with Borderline Dyslipidemia: A Randomized, Double-Blind, and Placebo-Controlled Clinical Trial

**DOI:** 10.1155/2016/9594251

**Published:** 2016-08-16

**Authors:** Hansongyi Lee, Hyerang Kim, Ryowon Choue, Hyunjung Lim

**Affiliations:** ^1^Department of Medical Nutrition, Graduate School of East-West Medical Science, Kyung Hee University, Yongin 17104, Republic of Korea; ^2^Research Institute of Medical Nutrition, Kyung Hee University, Seoul 02447, Republic of Korea

## Abstract

*Background.* Dyslipidemia has been well-known as a common metabolic disorder contributing to cardiovascular disease. The aim of this study was to evaluate the effect of the* Pinus koraiensis* needle extracts (PKE) on the blood cholesterol and oxidative stress.* Method.* We conducted a 12-week randomized, double-blinded controlled trial to examine the effect of PKE on blood lipid profiles in adults with borderline dyslipidemia. Thirty-three eligible persons were recruited and randomly assigned into PKE (*n* = 20) and placebo groups (*n* = 13). Serum lipids including total cholesterol, low-density lipoprotein- (LDL-) cholesterol, high-density lipoprotein- (HDL-) cholesterol, very low-density lipoprotein- (VLDL-) cholesterol, and triglyceride were measured before and after trial. Serum insulin, glucose, and antioxidant indicators were also analyzed before and after trial and anthropometry and blood pressure were measured every 4 weeks.* Results.* After 12 weeks, PKE statically significant decreases in systolic blood pressure (*p* < 0.05) and waist circumference (*p* < 0.05) were observed. Also, VLDL-cholesterol significantly decreased (from 24.4 ± 10.0 mg/dL at baseline to 18.4 ± 4.1 mg/dL after 12 weeks) (*p* < 0.05) and superoxide dismutase (SOD) increased (6.12 ± 0.41 U/mL to 9.06 ± 0.62 U/mL) (*p* < 0.01) in PKE group. However, after adjustment with WC, VLDL-cholesterol was not significant between groups (*p* = 0.095) and while SOD remained significant between groups (*p* = 0.013).* Conclusion.* The results show that PKE was effective in improving the superoxide dismutase in the individuals with borderline dyslipidemia.

## 1. Introduction

Cardiovascular disease (CVD) is one of the leading causes of death worldwide, estimating the incidence rate to above 17.3 million per year. The mortality rate from CVD has continuously increased from 2.3 persons in 1983 to 16.2 persons in 1998 and reached to 25.1 persons in 2012 per 10 million person in South Korea [1]. Dietary modification for prevention and management of chronic disease is not easy, and the natural complementary and alternative therapy as dietary supplements have been used.

*Pinus koraiensis, *which is known as Korean pine nuts, has long been used in traditional diet in many of Asian countries, such as Korea, Manchuria, and Japan. It is known to be a plentiful source of polyunsaturated fatty acids (PUFA) and monounsaturated fatty acids (MUFA), mostly trienoic acid and pinolenic acid [2]. Previous studies have shown the favorable effect of* Pinus koraiensis* on blood pressure [3], inflammatory response [4], and satiety and appetite control [5, 6]. However, the evidences regarding effects of blood lipid regulation of* Pinus koraiensis* are not still conclusive. Sugano et al. [3] examined the effect of* Pinus koraiensis* seed oil on blood lipids and they found that* Pinus koraiensis* seed oil specifically improved plasma triglyceride level and blood pressure compared to other similar materials like flaxseed oil. In some studies with similar study design, however, serum lipid profiles were not significantly changed [7, 8]. These controversial results in the experimental studies with animal model can be explained by handling with different methods of preparation and extraction, dose, and materials. However, it is hard to find clinical efficacy trials on* Pinus koraiensis* in human and besides it has seldom been examined the effect of* Pinus koraiensis* on the regulation of serum lipid profiles in the individuals with borderline dyslipidemia. Therefore, we examined the effect* Pinus koraiensis* needle extract (PKE) on lipid profiles and oxidative stress in the borderline dyslipidemia using a randomized and double-blinded controlled study design. This efficacy trial on PKE was also designed to provide clinical significance into the development of dietary supplementary products for early management of dyslipidemia.

## 2. Materials and Methods

### 2.1. Ethical Statement

The study protocol had been approved by the Kyung Hee University Hospital Ethics Committee (Seoul, South Korea) (IRB number: KMC IRB 1227-02). The trial was performed according to the Declaration of Helsinki and it was in accordance with the principles of Good Clinical Practice. All participants signed a written informed consent prior to study enrollment.

### 2.2. Participants

Inclusion criteria were (1) adult aged 20 or older and (2) borderline dyslipidemia (diagnosed with one of the abnormalities of serum lipid level as follows, 200–239 mg/dL of total cholesterol or 130–159 mg/dL of LDL-cholesterol or 150–199 mg/dL of triglyceride) [9]. We excluded individuals with any of the following conditions: (1) diagnosis of dyslipidemia and/or lipid lowering medication; (2) history and/or current treatment of chronic disease in cardiovascular system, kidney, and liver; (3) metabolic disturbances with thyroidal and pancreatic disease and diabetes; (4) alcoholics; (5) pregnancy and breastfeeding; (6) hormone replacement therapy; (7) unwillingness or inability to follow all trial procedures.

### 2.3. Sample Size Planning and Study Power

Sample size was estimated based on the results from a previous trial [10], where the detection of a significant difference between groups (*α* = 0.05 and *β* = 0.80) allowing for a 25% dropout rate suggested the recruitment of 35 people into each group.

### 2.4. PKE and Placebo Tablets

**Figure 1:**
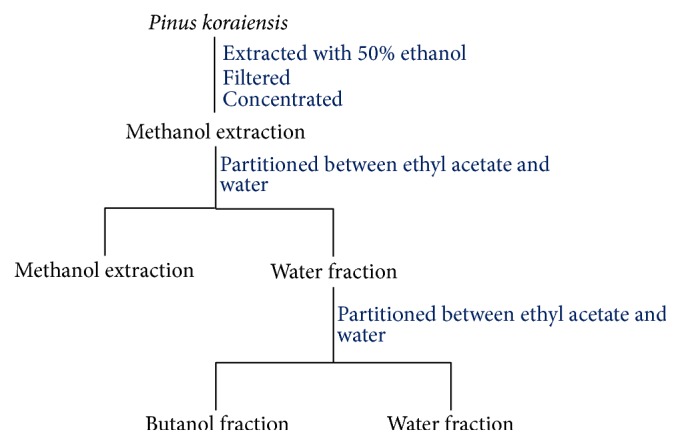
Flow diagram for extraction and fractionation of* Pinus koraiensis.*

The* Pinus koraiensis *needle was collected from natural pine stands in Hwacheon- myeon, Hongcheon-gun, Gangwon-do, Korea. The plant materials (200 g) were extracted with 50% ethanol with solvents as methanol (Figure 1). The extract was evaporated to an aqueous concentrate and then partitioned between ethyl acetate and water for 2 times at 45°C for 8 h. The extract was filtered through a 25 *μ*m standard sieve and was dried using a vacuum rotary evaporator to 60 Brix under low pressure, producing PKE tablets.

Each PKE tablet (450 mg) contained 300.0 mg PKE (66.6%) and various bulking agents, including sugar alcohol (12.4%), cellulose (10%), polysaccharide (2%), lubricating and glossing agents (5%), and other excipients (4%). The placebo tablets contained dextrin (66.6%) instead of PKE. All subjects ingested same number of PKE or placebo tablets, 2 tablets after morning and evening meals, for 12 weeks.

### 2.5. Study Design and Procedure

**Figure 2:**
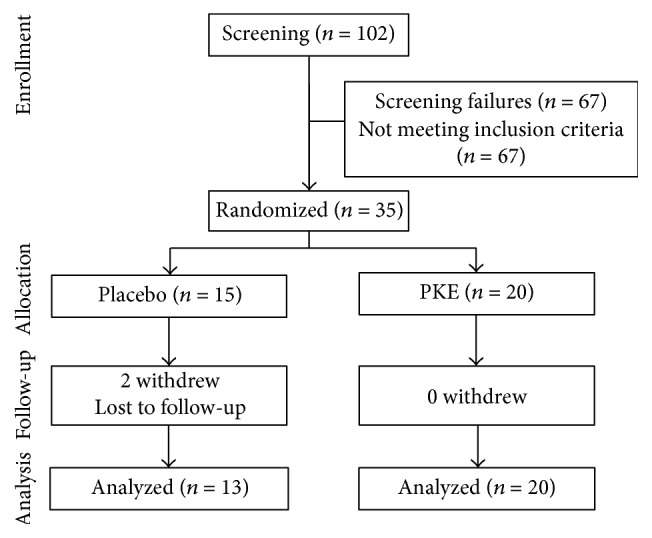
Subject's flow chart in Korean adults with borderline dyslipidemia in* Pinus koraiensis* extracts or placebo group for 12 weeks.

This study was randomized and double-blinded clinical trial design. Participants were recruited through a variety of outreach methods such as posters, word of mouth, presentation at community center, and website advertisements between October 2013 and January 2014 in Seoul, Korea. The persons who were interested in the study were briefly told about the purpose of the study, procedure, and the features of the products used in the study. Written consent form was obtained from the person with an affirmative response to participate into the study after further explanation of the study protocol. One hundred and two participants were interested in the study. Among them, only 35 subjects (enrollment rate: 34.3%) satisfied inclusion criteria through screening evaluation which confirmed their blood lipid profiles and other clinical statuses were enrolled in the 12-week clinical trial of PKE. The enrolled participants were randomly assigned to either test group (PKE group) or control group (Figure 2). Of the 35 subjects, two did not end in the placebo group, and hence there were 33 subjects for whom at least baseline data were collected and could be included in data analysis. After randomization, general information including age, sex, education, alcohol consumption, regular exercise, smoking status, disease checkup, and medication user was obtained using constructive survey questionnaire and anthropometrics and further laboratory measurement were conducted for baseline data collection.

### 2.6. Key Outcome Measures

Blood samples were drawn from the mid arm vein after 12-hour overnight fasting at baseline and at 12 weeks. Obtained samples were separated into ethylenediamine tetra-acetic acid-potassium (EDTA-K2) anticoagulant tubes and serum-separate tubes (SST). After being allowed to clot for 30 minutes, SST was centrifuged (3,000 ×g, 4°C, for 10 min) and the supernatant used for analysis. All laboratory analysis was conducted in clinical laboratory analysis institute named Green Cross Laboratories Co in Gyeonggi-do, Korea. Triacylglycerol (TG), total cholesterol, high-density lipoprotein- (HDL-) cholesterol, low-density lipoprotein- (LDL-) cholesterol, aspartic acid transaminase (AST), alanine transaminase (ALT), *γ*-glutamyl transpeptidase (*γ*-GT), and high sensitive C-reactive protein (hs-CRP) were analyzed using an automatic analysis system (Modular Analytics, Roche, Germany). Free-fatty acids were measured by a colorimetric method (ACS-ACOD) by means of a commercial assay kit (Roche, Germany). Very low-density lipoprotein- (VLDL-) cholesterol was estimated by the Friedewald equation: VLDL = triglyceride/5 [11]. Superoxide dismutase (SOD) was measured by colorimetric method using commercial kit (Superoxide Dismutase Assay Kit; Cayman, USA) (reference value: non), catalase was measured by spectrophotometer (UV1700, SHIMAZDA, Japan) using commercial kit (BIOXYTECH catalase-520; OxisResearch*™*, USA,) (reference value: 32.5–68.5 KU/L), and malondialdehyde (MDA) was measured by ELISA kit (OxiSelect*™* MDA Adduct; Cell Biolabs Inc., USA).

Anthropometric measures were conducted at baseline, 6 weeks, and 12 weeks. Body weight and height were measured with standard method, wearing light clothing and no shoes. Body mass index (BMI) was calculated as the ratio of weight (kg) to the square of height (m^2^). Waist circumference (WC) was assessed in the erect position at the middle between the tenth rib and the iliac crest, and hip circumference (HC) was assessed at the widest horizontal diameter of the buttock. Waist-to-hip ratio (WHR) was derived from the calculation with WC and HC. Systolic blood pressure (SBP) and diastolic blood pressure (DBP) were measured twice by mercury sphygmomanometer in the sitting position after they had been quietly seated for more than 15 min. The mean of the two measurements was used in the analyses.

### 2.7. Statistical Analysis

Descriptive analyses for continuous and discrete variables were presented as mean ± standard deviation (SD) or standard error (SE) and *n* (%), respectively. Nonparametric statistics such as Fisher's exact test and Mann-Whitney *U* test were used to compare differences in outcome variables between groups and Wilcoxon signed-rank test was used to assess within-group changes. General linear models (GLM) adjusted for WC the main effect and interactive effects of PKE. Efficacy analysis was conducted based on an intention-to-treat (ITT) principle [12]. All statistical analyses were performed using SPSS version 21.0 (IBM Cooperation, Chicago, USA). The significance level was defined at *p* < 0.05.

## 3. Results

General characteristics of subjects were presented in Table 1. The mean ages of the subjects were 46.1 ± 6.4 years in placebo group and 47.2 ± 7.5 years in PKE group. At baseline, there are no significant differences in BMI, WC, HC, WHR, SBP, DBP, smoking, alcohol consumption, and exercise between two groups.

After 12 weeks, WC were significantly decreased in both groups: −2.7 cm in decreased PKE (from 86.3 ± 9.8 cm at baseline to 84.8 ± 9.7 cm at after 12 weeks, *p* < 0.05) and −1.5 cm in decreased placebo group (from 88.2 ± 8.5 cm at baseline to 85.5 ± 9.3 cm after 12 weeks, *p* < 0.05). Also, SBP was significantly decreased −4.7 ± 8.9 mmHg (from 115.8 ± 11.8 mmHg at baseline to 110.3 ± 10.0 mmHg after 12 weeks, *p* < 0.05) in PKE group after 12 weeks but not in placebo group (from 115.6 ± 19.0 mmHg at baseline to 115.6 ± 14.1 mmHg at after 12 weeks) (data not shown) [Appendix].

Except for lipid profiles such as TG, TC, LDL, and FFA, VLDL-cholesterol level was significantly decreased −7.2 ± 5.3 mg/dL (from 25.0 ± 2.3 mg/dL at baseline to 18.5 ± 0.9 mg/dL after 12 weeks, *p* < 0.01) in PKE group; meanwhile, other indicators for lipid (TG, TC, LDL, and FFA) were not changed between and within groups after the intervention (Table 2). However, after adjustment with WC, VLDL-cholesterol was not significant between groups (*p* = 0.095).

For the results of antioxidative enzyme capacities (SOD and catalase) and lipid peroxidase (MDA) shown in Table 3, SOD was increased in both PKE (changes: 2.27 ± 0.62 U/mL from 6.12 to 9.06 ± 0.62 U/mL, *p* < 0.01) and placebo (changes: 2.30 ± 3.30 U/mL, from 8.84 to 9.06 ± 11.10 U/mL, *p* < 0.05) groups after 12 weeks. Changes in catalase and MDA were not detected. After adjustment with WC, SOD was significantly different between groups (*p* < 0.013).

## 4. Discussion

The primary objective of this study was to examine the PKE on lipid lowering in Korea adults with borderline dyslipidemia. The results showed a modest but favorable difference in total serum cholesterol, although VLDL-cholesterol showed statistically significant improvement in PKE group. Additionally, antioxidant levels, as a secondary outcome, were not significantly different between groups after supplement, although SOD was positive supplement effect. On the other results, the WC and SBP were changed in PKE group after supplement. After adjustment with WC, VLDL-cholesterol was not significant between the groups, while SOD remained significant between groups. Also, lack of any significant effect of the PKE on blood levels of AST and ALT compared with the placebo group demonstrated that* Pinus koraiensis *does not have a toxic effect on the hepatic function. Moreover, no adverse effects were reported by the subjects.

*Pinus* contains about 15% of polyunsaturated fatty acid (PUFA) known as pinolenic acid (cis-5, 9, 12-18:3). Pinolenic acid contributes to triacylglycerol-lowering properties such as decreased de novo lipid synthesis, reduced substrate availability for lipoprotein formation, or changes in VLDL physicochemical properties [13]. Therefore,* Pinus koraiensis* may reduce triacylglycerol and VLDL concentration and may have a potential benefit in lowering CVD risk. In accordance with Sugano et al. [3], observed levels of serum triglycerides in rats supplemented with* Pinus koraiensis* seed oil (10 g/kg B.W/day corresponding to 1.8 g pinolenic acid/kg B.W/day) for 5 weeks showed a hypocholesterolemic effect. Similarly, Asset [7] showed the* Pinus koraiensis* seed oil (5 g/kg B.W/day corresponding to 0.75 g pinolenic acid/kg B.W/day) for 4 weeks lowering triglycerides serum triglycerides by 16% and VLDL-triglycerides by 21%. Earlier, Wolff [14] showed* P. pinaster,* kinds of* pine*, seed oil lowers triglycerides, VLDL-triglycerides, and VLDL-cholesterol compared to a diet enriched in oleic acid. Also, Kim et al. [15] showed that the* Pinus koraiensis* was upregulated LDL-receptor as well as being negative and HMG-CoA reductase. The removal of the LDL-cholesterol from blood is mainly mediated by receptor-dependent process.

In our study, despite PKE supplementation, TG and TC were, respectively, 8.2% and 3.8% decreased, and data were not significant. We thought that the subjects were mainly with cholesterol borderline, rather than with dyslipidemia individuals with >240 mg/dL of serum total cholesterol or >160 mg/dL of LDL-cholesterol or >200 mg/dL of triglyceride and sample size was small. These lead to a decrease power in detecting differences between groups. Also, HDL-cholesterol was decreased in PKE group; in our group, there was not mechanistic study on why HDL-cholesterol was decreased; however, previously, one study shows that* pine *supplementation decreased in HDL-cholesterol, and further study will be needed.

Our findings are also as follows: PKE reduced SBP by −4.7 mmHg after 12 weeks in PKE group. In accordance with animal data, the 8 weeks of the pine seed oil supplement (1.8 g pinolenic acid/kg body weight/day) alleviated SBP in spontaneously hypertensive rat (9 weeks old male rats) [3]. This mechanism may explain that pinolenic acid attenuated blood pressure through the imbalance of prostaglandins, the influencing factor of the blood pressure [3, 16].

Recently, Ko and colleges [17] showed that the essential oil of* Pinus koraiensis* effects suppressed body weight gain though may decrease the expression peroxisome proliferator-activated receptor (PPAR). In our study, WC was decreased in PKE group compared with placebo group, although body weight was not changed. WC and WHR have been suggested to be strongly associated with metabolic risk factors because of being inversely associated with dyslipidemia, diabetes, hypertension, and CVD.

Several limitations of our study have to be addressed like low success for recruitment to the intervention. In our study, inclusion criteria were stricter than that of other studies, namely, difficulty in screening and higher screening failure rate (65%). It has affected the sample size for this study which was smaller than planned sample size (each group equals 35). These two limitations affected a major cause between groups which was not significant. Despite these limitations, our study showed excellent compliance among the 33 subjects (placebo: 94.2% and PKE group: 91.7%) and without any discomfort.

In conclusion, the present study is the first to report that PKE could be of beneficial effect as VLDL-cholesterol decreased and SOD increased. However, after adjustment with WC, VLDL-cholesterol was not significant between groups, while SOD remained significant. Further studies are needed to determine difference for the lipid profiles with enough sample size. In our results, PKE also reduced WC and SBP after 12 weeks of consumption without any adverse effects. Based on our results, future studies should examine PKE effects on the usefulness for subject with dyslipidemia or metabolic syndrome.

## Figures and Tables

**Figure 3:**
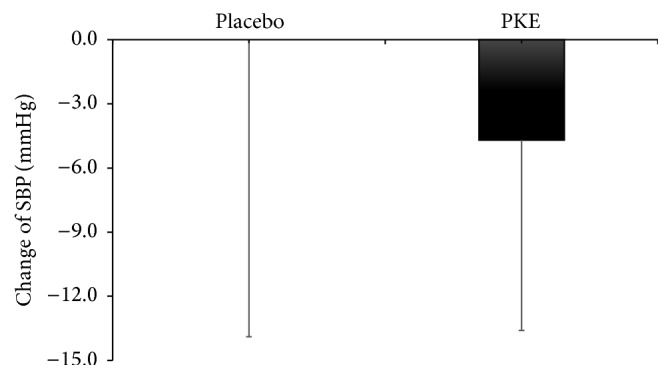
Changes of the SBP in the* Pinus koraiensis* or placebo group for 12 weeks. No significant difference between the two groups by Student's* t*-test.

**Table 1:** Anthropo- and health-related subjects by treatment group at the baseline.

	Placebo (*n* = 13)	PKE (*n* = 20)
Age (years)	46.1 ± 6.4^(1)^	47.2 ± 7.5
Sex, M/F (*n*, %)	5 (38.5)/8 (61.5)	6 (30.0)/14 (70.0)

Body mass index (kg/m^2^)	25.1 ± 4.2	24.2 ± 3.6
Waist circumference (cm)	88.2 ± 8.5	86.3 ± 9.8
Waist-to-hip ratio	0.88 ± 0.04	0.89 ± 0.05
Systolic blood pressure (mmHg)	115.6 ± 19.0	115.8 ± 11.8
Diastolic blood pressure (mmHg)	81.7 ± 13.2	79.9 ± 9.6

Smoking (*n*, %)	3 (23.1)	2 (10.0)
Cigarette/day	10.8 ± 8.8	9.75 ± 7.4
Alcohol consumption (*n*, %)	10 (76.9)	14 (70.0)
Amount (g/week)	75.3 ± 82.2	49.4 ± 79.5
Exercise regularly (*n*, %)	8 (61.5)	9 (45.0)
Duration (min/week)	182.5 ± 165.0	240.6 ± 123.1

^(1)^Values are mean ± SD or *n* (%).

No significant difference between the two groups by Student's *t*-test.

**Table 2:** Blood lipid profiles before and after intervention, by treatment group.

	Placebo (*n* = 13)		PKE (*n* = 20)	
	Before	After	Before	After
Aspartic acid transaminase (U/L)	24.2 ± 3.0^(1)^	20.5 ± 1.3	23.1 ± 1.9	21.6 ± 1.1
Alanine transaminase (U/L)	20.8 ± 4.0	16.5 ± 2.6	21.0 ± 2.5	21.9 ± 3.1
*γ*-glutamyl transferase (U/L)	28.0 ± 10.7	25.1 ± 6.7	24.0 ± 3.8	23.9 ± 4.0

Triglyceride (mg/dL)	137.3 ± 38.3	110.5 ± 17.9	110.6 ± 9.6	92.5 ± 4.4
Total cholesterol (mg/dL)	205.4 ± 7.8	198.7 ± 8.4	205.9 ± 5.4	197.1 ± 5.7
LDL-cholesterol (mg/dL)	135.7 ± 6.4	130.8 ± 6.9	134.0 ± 4.7	131.5 ± 4.5
HDL-cholesterol (mg/dL)	50.2 ± 3.2	54.7 ± 3.1	59.4 ± 3.0^*∗*^	55.4 ± 3.5
VLDL-cholesterol (mg/dL)^(2)^	29.3 ± 8.0	22.2 ± 3.6	25.0 ± 2.3	18.5 ± 0.9^††^
Free-fatty acid (*μ*Eq/L)	497.5 ± 90.1	453.7 ± 61.0	496.6 ± 48.2	500.7 ± 30.9

Glucose (mg/dL)	94.6 ± 3.9	95.8 ± 3.1	94.6 ± 2.0	92.3 ± 1.6
Insulin (*μ*IU/mL)	7.9 ± 1.7	5.5 ± 0.8	7.4 ± 0.8	6.6 ± 0.9
QUICKI^(3)^	0.36 ± 0.01	0.38 ± 0.01	0.36 ± 0.01	0.37 ± 0.01^†^

^(1)^ Data are mean ± SE.

^(2)^VLDL-cholesterol calculation: triglyceride/5.

^(3)^QUICKI: quantitative insulin-sensitivity check index: 1/[log (fasting insulin) + log (fasting glucose, mg/dL)].

^*∗*^Significant difference between the two groups by Student's *t*-test at ^*∗*^ *p* < 0.05.

^†^Significant difference within groups by paired *t*-test at ^†^ *p* < 0.05 and ^††^ *p* < 0.01.

**Table 3:** Antioxidants profiles before and after intervention, by treatment group.

	Placebo (*n* = 13)		PKE (*n* = 20)	
	Before	After	Before	After
Superoxide dismutase (U/mL)	8.84 ± 1.9^(1)^	11.10 ± 1.8^†^	6.12 ± 0.41	9.06 ± 0.62^††^
Catalase (KU/L)	7.72 ± 5.1	6.83 ± 1.2	5.10 ± 1.89	6.46 ± 1.2
Malondialdehyde (pmol/mg)	0.88 ± 0.1	0.81 ± 0.1	1.02 ± 0.08	0.84 ± 0.06

^(1)^Data are mean ± SE.

^†^Significant difference within groups by paired *t*-test at ^†^ *p* < 0.05 and ^††^ *p* < 0.01.

**Table 4:** Anthropometric measurements of the subjects.

	Placebo (*n* = 13)		PKE (*n* = 20)	
	Before	After	Before	After
Height (cm)	164.5 ± 7.7^(1)^		161.4 ± 7.1	
Weight (kg)	68.5 ± 14.6	67.7 ± 14.0	63.4 ± 14.0	63.3 ± 14.0
Body mass index (kg/m^2^)	25.1 ± 4.2	24.9 ± 4.0	24.2 ± 3.6	23.1 ± 5.6
Waist circumference (cm)	88.2 ± 8.5	85.5 ± 9.3^†^	86.3 ± 9.8	84.8 ± 9.7^†^
Hip circumference (cm)	99.7 ± 6.7	98.6 ± 7.4^†^	96.6 ± 6.3	96.0 ± 6.0
Waist-to-hip ratio	0.88 ± 0.04	0.87 ± 0.04	0.89 ± 0.05	0.88 ± 0.05
Systolic blood pressure (mmHg)	115.6 ± 19.0	115.6 ± 14.1	115.8 ± 11.8	110.3 ± 10.0^*∗*†^
Diastolic blood pressure (mmHg)	81.7 ± 13.2	79.3 ± 13.2	79.9 ± 9.6	79.1 ± 8.8

^(1)^Data are mean ± SD.

^*∗*^Significant difference between the two groups by Student's *t*-test.

^†^Significant difference within groups by paired *t*-test *p* < 0.05.

## Appendix

See Table 4 and Figure 3.

## References

[1] Korea National Statistical Office (2013). *Korea National Statistical Office Cause of Death Statistics in 1983–2012*.

[2] Wolff R. L., Bayard C. C. (1995). Fatty acid composition of some pine seed oils. *Journal of the American Oil Chemists' Society*.

[3] Sugano M., Ikeda I., Wakamatsu K., Oka T. (1994). Influence of Korean pine (Pinus koraiensis)-seed oil containing cis-5,cis-9,cis-12-octadecatrienoic acid on polyunsaturated fatty acid metabolism, eicosanoid production and blood pressure of rats. *British Journal of Nutrition*.

[4] Park S., Lim Y., Shin S., Han S. N. (2013). Impact of Korean pine nut oil on weight gain and immune responses in high-fat diet-induced obese mice. *Nutrition Research and Practice*.

[5] Pasman W. J., Heimerikx J., Rubingh C. M. (2008). The effect of Korean pine nut oil on in vitro CCK release, on appetite sensations and on gut hormones in post-menopausal overweight women. *Lipids in Health and Disease*.

[6] Hughes G. M., Boyland E. J., Williams N. J. (2008). The effect of Korean pine nut oil (PinnoThin*™*) on food intake, feeding behaviour and appetite: a double-blind placebo-controlled trial. *Lipids in Health and Disease*.

[7] Asset G., Baugé E., Wolff R. L. (1999). Effects of *Pinus pinaster* and *Pinus koraiensis* seed oil supplementation on lipoprotein metabolism in the rat. *Lipids*.

[8] Won S. B., Jung G.-Y., Kim J., Chung Y. S., Hong E. K., Kwon Y. H. (2013). Protective effect of pinus koraiensis needle water extract against oxidative stress in HepG2 cells and obese mice. *Journal of Medicinal Food*.

[9] Korea Society of Lipidology and Atherosclerosis

[10] Kianbakht S., Abasi B., Perham M., Hashem Dabaghian F. (2011). Antihyperlipidemic effects of *Salvia officinalis L.* leaf extract in patients with hyperlipidemia: a randomized double-blind placebo-controlled clinical trial. *Phytotherapy Research*.

[11] Friedewald W. T., Levy R. I., Fredrickson D. S. (1972). Estimation of the concentration of low-density lipoprotein cholesterol in plasma, without use of the preparative ultracentrifuge.. *Clinical Chemistry*.

[12] Hollis S., Campbell F. (1999). What is meant by intention to treat analysis? Survey of published randomised controlled trials. *British Medical Journal*.

[13] No D. S., Kim I.-H. (2013). Pinolenic acid as a new source of phyto-polyunsaturated fatty acid. *Lipid Technology*.

[14] Wolff R. L. (1995). Structural importance of the cis-5 ethylenic bond in the endogenous desaturation product of dietary elaidic acid, cis-5, trans-9 18 : 2 acid, for the acylation of rat mitochondria phosphatidylinositol. *Lipids*.

[15] Kim J.-H., Lee H.-J., Jeong S.-J., Lee M.-H., Kim S.-H. (2012). Essential oil of pinus koraiensis leaves exerts antihyperlipidemic effects via up-regulation of low-density lipoprotein receptor and inhibition of acyl-coenzyme A: cholesterol acyltransferase. *Phytotherapy Research*.

[16] Knapp H. R. (1989). Omega-3 fatty acids, endogenous prostaglandins, and blood pressure regulation in humans. *Nutrition Reviews*.

[17] Ko H.-S., Lee H.-J., Lee H.-J. (2013). Essential oil of *Pinus koraiensis* exerts antiobesic and hypolipidemic activity via inhibition of peroxisome proliferator-activated receptors gamma signaling. *Evidence-Based Complementary and Alternative Medicine*.

